# New Insights into Development of Female Reproductive Tract—Hedgehog-Signal Response in Wolffian Tissues Directly Contributes to Uterus Development

**DOI:** 10.3390/ijms22031211

**Published:** 2021-01-26

**Authors:** Ryuma Haraguchi, Gen Yamada, Aki Murashima, Daisuke Matsumaru, Riko Kitazawa, Sohei Kitazawa

**Affiliations:** 1 Department of Molecular Pathology, Ehime University Graduate School of Medicine, Shitsukawa, Toon City, Ehime 791-0295, Japan; riko@m.ehime-u.ac.jp (R.K.); kitazawa@m.ehime-u.ac.jp (S.K.); 2 Department of Developmental Genetics, Wakayama Medical University, Kimiidera 811-1, Wakayama City, Wakayama 641-8509, Japan; transg8@wakayama-med.ac.jp (G.Y.); murashimamura930@gmail.com (A.M.); matsumaru-da@gifu-pu.ac.jp (D.M.); 3 Department of Anatomy, Iwate Medical University, Idaidori 1-1-1, Yahaba, Iwate 028-3694, Japan; 4 Laboratory of Hygienic Chemistry and Molecular Toxicology, Gifu Pharmaceutical University, Daigaku-nishi 1-25-4, Gifu City, Gifu 501-1196, Japan; 5 Division of Diagnostic Pathology, Ehime University Hospital, Shitsukawa, Toon City, Ehime 791-0295, Japan

**Keywords:** female reproductive tract, Wolffian ducts, Mullerian ducts, hedgehog signaling, genetic lineage tracing

## Abstract

The reproductive tract in mammals emerges from two ductal systems during embryogenesis: Wolffian ducts (WDs) and Mullerian ducts (MDs). Most of the female reproductive tract (FRT) including the oviducts, uterine horn and cervix, originate from MDs. It is widely accepted that the formation of MDs depends on the preformed WDs within the urogenital primordia. Here, we found that the WD mesenchyme under the regulation of Hedgehog (Hh) signaling is closely related to the developmental processes of the FRT during embryonic and postnatal periods. Deficiency of Sonic hedgehog (Shh), the only Hh ligand expressed exclusively in WDs, prevents the MD mesenchyme from affecting uterine growth along the radial axis. The in vivo cell tracking approach revealed that after WD regression, distinct cells responding to WD-derived Hh signal continue to exist in the developing FRT and gradually contribute to the formation of various tissues such as smooth muscle, endometrial stroma and vascular vessel, in the mouse uterus. Our study thus provides a novel developmental mechanism of FRT relying on WD.

## 1. Introduction

The female reproductive tract (FRT) originates from the Mullerian ducts (MDs) that contribute to the formation of the oviducts, uterine horn and cervix [1,2,3]. During embryogenesis, the formation of MDs depends on preformed Wolffian ducts (WDs) within the urogenital ridge [4,5]. The WD, also called the mesonephric duct, emerges from the intermediate mesoderm and elongates caudally until it reaches the cloaca. Following WD formation, cells of the coelomic epithelium, a single layer of cells covering the entire body cavity, is specified to MD precursors by WD-derived inductive signals; MD precursors then invaginate by linking with these canalizations and elongate caudally toward the urogenital sinus using WDs as “guide wires” [5].

Interactions between WDs and MDs are assumed to play roles in FRT formation because WDs localize close to the coelomic epithelium or MDs during embryogenesis. In practice, several studies have shown that the WD is essential for early MD morphogenesis during FRT formation [6,7,8,9]. The functional roles of WDs during the initiation of MDs have been described. Namely, Wolffian-derived signals coupled with the BMP/Pax2 axis activate FGF/ERK signaling. Subsequently, *Lim1* expression, a hallmark of MD specification and invagination, is induced in the coelomic epithelium [6]. Mouse genetic studies have also shown a strong dependency of MD elongation processes on the WD. The WD-specific targeted ablation of the *Lim1* gene elicits WD degeneration and severe MD defects causing impairment of MD caudal elongation toward the urogenital sinus [9]. Similarly, physical elimination of WDs in female mice with the use of the Diphtheria Toxin-mediated genetic cell ablation system exhibits agenesis of the uterus [7,8], which is caused by a lack in the guidance ability of WDs for the elongation of caudal MDs. Thus, the previous studies indicate that WDs play a primary role as a morphogenetic machinery that controls the early phase of MD formation, initiation and elongation processes in FRT development.

In mammals, after the completion of MD elongation, the radial growth and differentiation of uterine stroma are gradually initiated under the influence of various growth factors and hormonal regulations through the interaction between the MD epithelium and its surrounding mesenchyme [10,11,12]. At the late embryonic stage, although the WD starts to regress by the lack of testicular testosterone production in the female, it continues to exist near the MDs until birth meanwhile uterine growth along the radial axis is observed [13]. Due to these positioning aspects of the two ductal systems, functional roles of WDs are also anticipated after the establishment of MDs. Nonetheless, developmental implications of the regressing WD in females at the late phase of MD morphogenesis remain unknown.

The Hedgehog (Hh) signaling pathway plays multiple roles in the development of the female reproductive organs [14]. Among three Hh ligands, Sonic hedgehog (Shh) is expressed in the WD epithelium during early embryogenesis [15,16]. *Patch1*, the main receptor for Hh ligands, and *Gli1*, a transmitter of Hh intracellular signaling, are expressed in urogenital tissues near WDs, implying a paracrine interaction between WDs and surrounding cells driven by a Shh-Ptch1 axis [17]. Although the components of the Hh signaling pathway are present in the Wolffian region, there is, however, no functional data that allude to their role in the developing FRT.

Here, the implication of the WD-derived Hh signal in the formation of the FRT was examined through mouse genetic analyses. In the absence of Shh function, although the mutant uterus showed no obvious defects on the elongation of MDs, its radial growth was markedly impaired. Irreversible genetic marking based on the mouse Cre-loxP system revealed that Hh-signal-responsive progenitors located in the urogenital mesenchyme surrounding WDs can contribute to various tissue components of the uterus. These results newly elucidate a potential role for WD-derived Hh in the FRT development.

## 2. Results

### 2.1. Characterization of Hedgehog-Related Gene Expression in Urogenital Ridge Region

**Figure 1:**
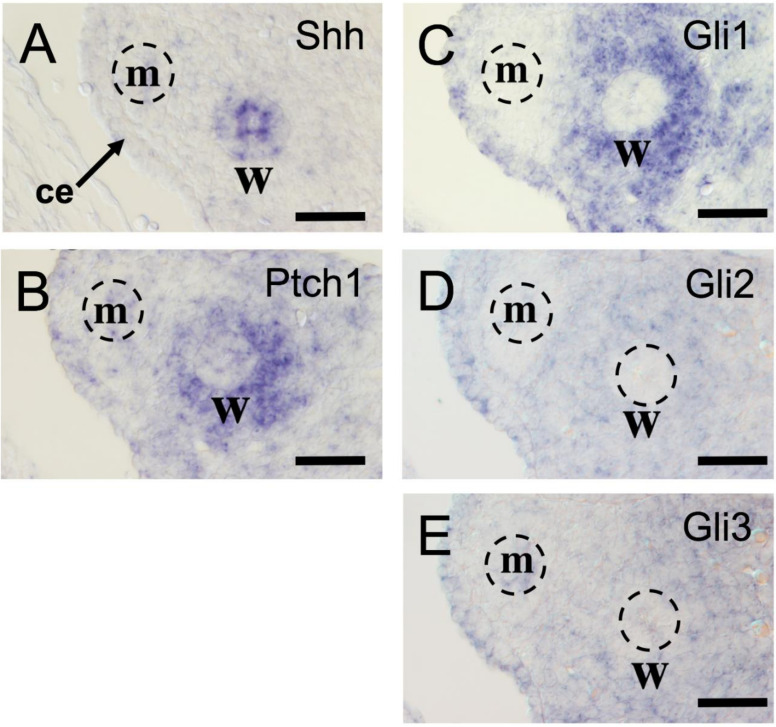
Expression pattern of Hh signaling genes in embryonic reproductive tracts. (**A**) At E13.5, *Shh* mRNA expression is observed in the Wolffian duct. (**B**,**C**) *Ptch1* and *Gli1* mRNA expression is observed specifically in the mesenchymal cells surrounding Wolffian duct. (**D**,**E**) *Gli2* and *Gli3* are weakly and diffusely expressed in the mesonephric region. Ce, coelomic epithelium; m, Mullerian duct; w, Wolffian duct. Scale bars indicate 50 μm.

In mice, the paired WDs and MDs are clearly observed within the urogenital ridges until E14.5, and then WDs gradually regress in female embryos. To establish the spatial expression profile of Hh-related genes at the Wolffian region during the formation of the FRT, the expression of *Shh*, *Ptch1*, *Gli1*, *Gli2*, and *Gli3* was examined in the urogenital ridge of female embryos at E13.5. *Shh* mRNA was detected in the WD epithelium (Figure 1A), as described in previous studies [15,16]. The expression of both *Ptch1* and *Gli1* genes was observed in the mesenchyme adjacent to WDs and was not observed in the tissue around MDs (Figure 1B,C). *Gli2* and *Gli3* mRNA were weakly expressed throughout the urogenital ridge mesenchyme (Figure 1D,E). No obvious sexual dimorphic pattern of the expression of these genes was observed in the urogenital ridge at this stage.

### 2.2. Developmental Defects of Female Reproductive Tract in Shh KO Mice

**Figure 2:**
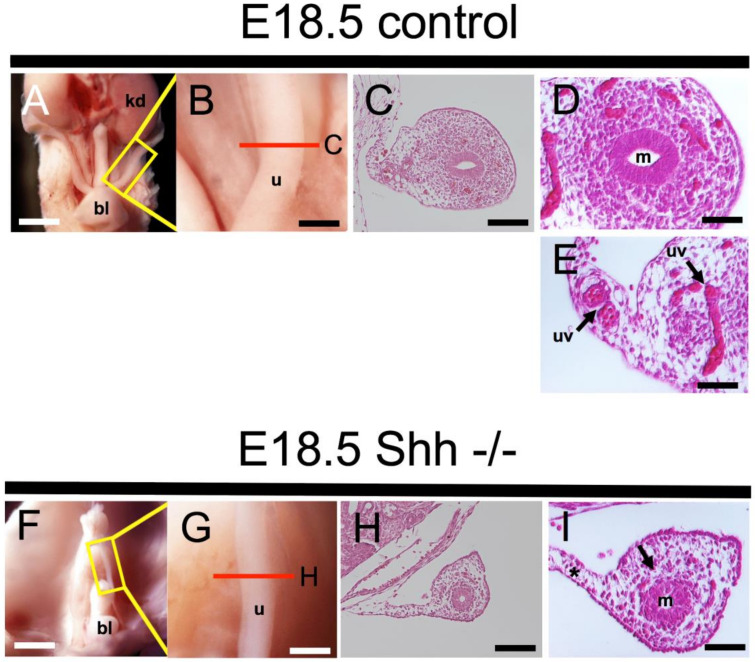
Developmental defects of FRT in *Shh* KO mice. (**A**,**B**,**F**,**G**) Macroscopic frontal views of E18.5 control and *Shh* mutant urogenital organs. Mutants show severe developmental anomalies in the bladder, kidney and uterus (**F**,**G**). (**C**–**E**,**H**,**I**) Section images of HE-stained control and mutant uterus. In (**I**), mutants indicate small amounts of mesenchymal tissue surrounding the Mullerian duct (black arrow) and the lack of uterine artery in the uterus (asterisk). Red lines in (**B**) and (**G**) indicate sites of transverse sections in (**C**,**H**). (**D**,**E**,**I**) Higher magnification of control and mutant uterus. These results were observed in several individuals (*n* = 3). bl, bladder; kd, kidney; u, uterus; uv, uterine vessel; m, Mullerian duct. Scale bars indicate 500 μm (**A**,**F**), 200 μm (**B**,**G**), 100 μm (**C**,**H**), and 50 μm (**D**,**E**,**I**).

The FRT originates from MDs whose development is definitely dependent on WDs, indicating that WDs loss or degeneration causes severe developmental defects in Mullerian derivatives including the oviduct, uterus, cervix, and upper vagina [3,7,9,18]. The components of the Hh signaling pathway were detected in the Wolffian region during FRT formation (Figure 1). To determine the effects of Hh signaling on FRT development, *Shh* KO mice were analyzed at E18.5 (Figure 2). The urinary tracts of mutants were markedly disorganized, showing severe hypoplasia of the kidney and the bladder (Figure 2F), as described [19,20]. In reproductive organs, *Shh* KO females exhibited anomalies in MD derivatives. The mutant uterus displayed a severely reduced length as compared with the control uterus (Figure 2A,F); also observed was distinct uterine dysplasia. Although the mutant uterus contained MDs that were barely recognizable as tubular structures (Figure 2H), they showed a prominent reduction in the mesenchymal mass surrounding the MD epithelium and agenesis of uterine vessels on the mesometrial side of the uterus (Figure 2I). These mesenchymal and vascular defects of uterus in *Shh* KO mice were validated by immunostaining of E-cadherin, CD31 and ERα (Figure S2). These data suggest that Shh is essential for the stromal components of the embryonic uterus, and that deficiency of Shh activity in the female embryo causes dysplasia.

### 2.3. Wolffian Region Specific Hedgehog-Signal-Responsiveness in Embryonic Reproductive Tract

**Figure 3:**
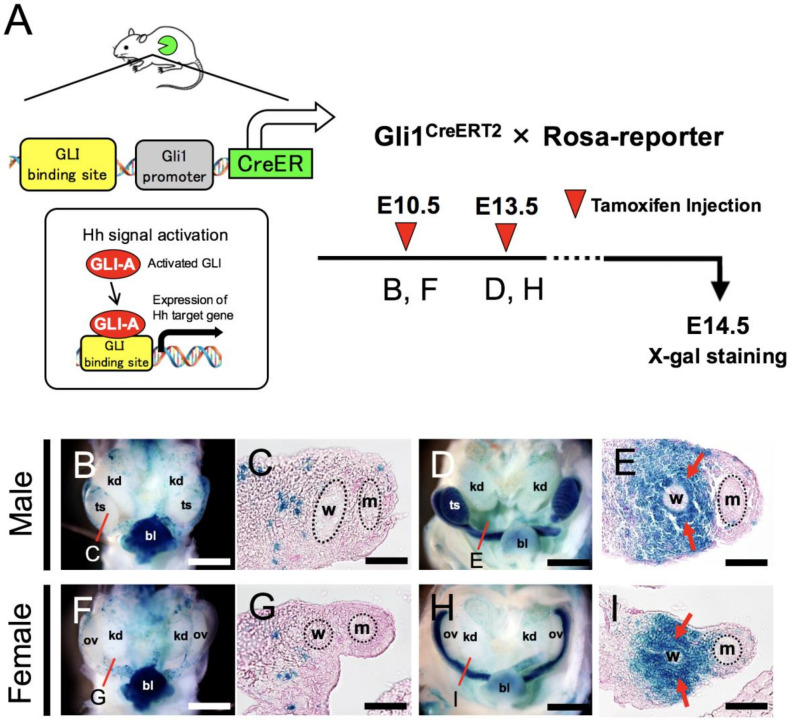
*Gli1^CreERT2^* allele marks Hh-signal-responsive cells in the Wolffian region. (**A**) The left panel shows a schema of the transcriptional machinery of the *Gli1*^CreERT2^ allele. In the Hedgehog (Hh) pathway-activated cells, the *Gli1*^CreERT2^ allele expresses a tamoxifen (TM) inducible Cre recombinase inserted into the *Gli1* locus, a Hh direct target gene. The right panel shows the experimental schedule for TM administration used in (**B**–**I**) to analyze the contribution of Hh-signal-responsive cells to the reproductive tissue formation during the embryonic stage. (**B**–**I**) Whole-mount and section images of X-gal-stained *Gli1*^CreERT2^; *Rosa26*-LacZ specimens at E14.5. Red lines in (**B**,**D**,**F**,**H**) indicate sites of transverse sections in (**C**,**G**,**E**,**I**). In both male and female embryos administered TM at E13.5, β-galactosidase activity showing Hh-signal-responsiveness (red arrows) is detectable in the mesenchymal cells surrounding the WD (**E**,**I**). Compared with E13.5 labeling, X-gal-stained cells in E10.5 TM injected embryos are sparsely scattered in the Wolffian tissues (**C**,**G**). These results were observed in several individuals (*n* = 3). bl, bladder; kd, kidney; md, Mullerian duct; ov, ovary; ts, testis; w, Wolffian duct. Scale bars indicate 200 μm (**B**,**F**,**D**,**H**) and 50 μm (**C**,**E**,**G**,**I**).

*Shh* is specifically expressed at WDs within the urogenital ridge, and *Shh* KO female mutants display the developmental defects of uterus (Figure 1 and Figure 2). This suggests that the responsiveness of FRT progenitor cells to Hh signaling may play roles in the developmental events of the embryonic uterus. To acquire clues to elucidate the action of Hh signaling during FRT formation, fate mapping of Hh-signal responsive cells in developing urogenital tissues was carried out. A Hh signal-specific Cre-driver line, *Gli1*^CreERT2^ mouse strain [21], was used to reveal Hh-responsiveness. The *Gli1* gene is a well-established Hh signaling target and effector gene [22,23,24], and *Gli1*^CreERT2^ mice express Tamoxifen (TM)-inducible Cre recombinase from the endogenous *Gli1* locus in response to Hh stimulation (Figure 3A). When crossed with the *Rosa26R* Cre indicator strain, *Gli1*^CreERT2^-positive cells exposed to TM are genetically labeled by permanent reporter gene expression based on Cre–*lox*P recombination in the Rosa26 locus. Thus, the reporter-labeled Hh-responsive cells and their progeny can be detected and tracked for various periods of time (Figure 3A). To investigate Hh-responsiveness in the urogenital ridge during the formation of reproductive tracts, pregnant females bearing *Gli1*^CreERT2^; *Rosa26-LacZ* embryos were treated with TM on days 10.5 or 13.5 of gestation, and then both male and female embryos were examined for the presence of LacZ positive cells at E14.5 (Figure 3A). By TM treatment at E10.5, the period for forming WDs and their elongation caudally to the cloaca, weakly and diffusely labeled cells were observed in the mesenchymal tissues of the urogenital ridge at E14.5 in both male and female *Gli1*^CreERT2^; *Rosa26-LacZ* mice (Figure 3B,C,F,G). When TM was administered at E13.5, a period completing the formation of MDs along the WDs, strong LacZ positive signals were detected in developing reproductive tracts along the rostrocaudal axis in males and females at E14.5 (Figure 3D,H). In the male gonad, β-galactosidase activity was clearly observed as reported [25,26] (Figure 3D); by contrast, the female gonad showed no obvious β-galactosidase activity (Figure 3H). In the mesonephric region, histological analysis indicated high staining intensity of specific β-galactosidase activity in the mesenchymal tissues surrounding the WDs with no obvious differences between male and female embryos (Figure 3E,I). This distribution pattern of reporter-labeled cell after TM induction at E13.5 was similar to the expression pattern of *Gli1* gene at the same stage as described above (Figure 1). These results indicated that Shh derived from the WDs is received by the mesenchyme surrounding the WDs in a paracrine manner, but not by the WDs-epithelial cells, and not by the peri-Mullerian mesenchyme.

### 2.4. Contribution of Wolffian-Derived Hedgehog-Signal-Responsive Cells to Developing and Adult Uterine Tissues

**Figure 4:**
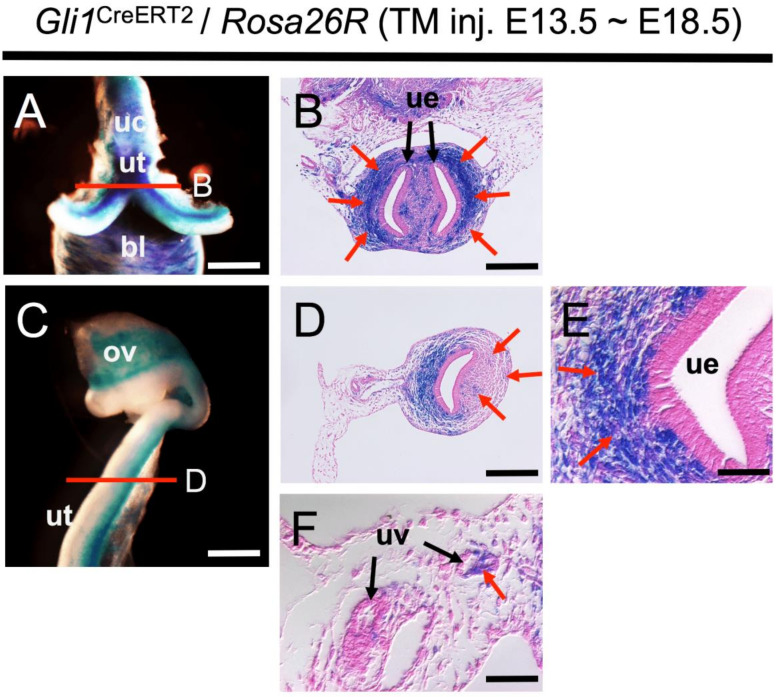
Developmental contribution of Hh-signal-responsive cells in the Wolffian region to embryonic uterus. (**A**–**F**) Whole-mount and section images of X-gal-stained *Gli1*^CreERT2^; *Rosa26*-LacZ female specimens at E18.5. Red lines in (**A**) and (**C**) indicate sites of transverse sections in (**B**,**D**). (**E**,**F**) Higher magnification of the uterine epithelium and uterine vessels on the mesometrial side. (**B**,**D**–**F**) TM administration at E13.5, β-galactosidase activity (red arrows) showing Hedgehog-responsiveness is mainly observed in mesenchymal cells surrounding the mesometrial side of uterine epithelia at E18.5. A few LacZ-positive cells are observed in the antimesometrial side (**D**, red arrows). These results were observed in several individuals (*n* = 3). bl, bladder; ov, ovary; uv, uterine vessel; uc; uterine cervix; ue, uterine epithelia; ut, uterus. Scale bars indicate 500 μm (**A**), 200 μm (**C**), 100 μm (**B**,**D**), and 50 μm (**E**,**F**).

In embryonic reproductive tract tissues, as with males, females also show strong response to the Hh signal produced from WDs (Figure 3). To determine the possible role of such Hh-responsive cells in uterine development, long-term lineage tracing was conducted. TM was administered at E13.5, a period of active reporter-labeling around WD tissues, and *Gli1*^CreERT2^; *Rosa26*-LacZ female embryos were examined 5 days thereafter (Figure 4). The gross appearance of the urogenital organs after TM induction clearly revealed Hh-responsive Wolffian-derived cells throughout the FRT, such as ovary, oviduct, uterus, and cervix, along the rostrocaudal axis (Figure 4A,C). Histological observations revealed highly localized β-galactosidase activity and reception of Hh signaling in the mouse developing uterus (Figure 4B,D–F). Interestingly, most reporter-labeled cells were detected mainly in the mesenchyme surrounding the uterine epithelium (Figure 4B,E) and in uterine vessels on the mesometrial side (Figure 4F). Additionally, small clusters of LacZ-positive cell were observed on the antimesometrial side (Figure 4D). The mesometrium constitutes the majority of the broad ligament of the uterus; in mice from mid to late gestation, it is gradually constructed on the inner side against MDs of the urogenital ridge, where WDs are located. Taking into account the results of E13.5 fate mapping and the histoanatomical aspects of the developing FRT, we assume that Wolffian Hh-responding cells function specifically as critical developmental tissue components of the uterine mesometrial pole.

**Figure 5:**
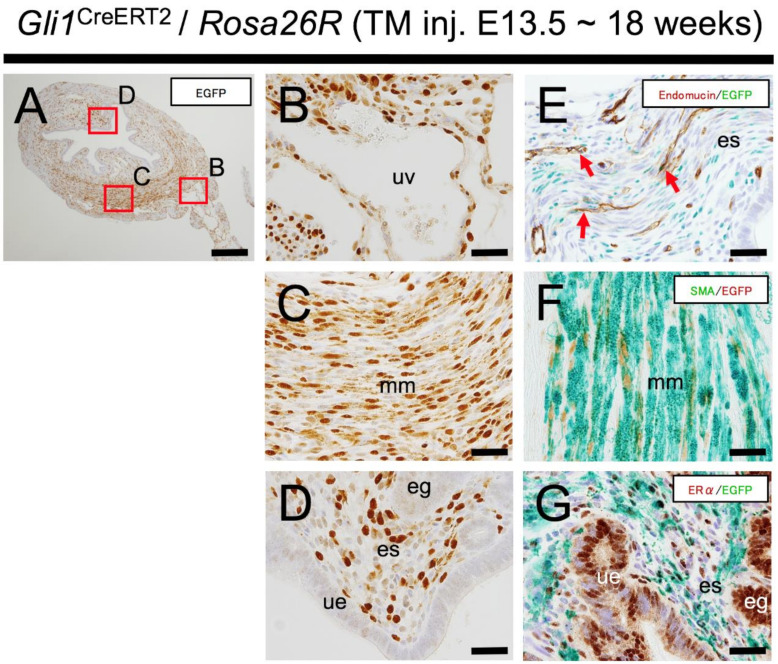
Developmental contribution of Hh-signal-responsive cells in the Wolffian region to adult uterus. (**A**–**D**) Immunohistostaining with anti-EGFP Ab of the mouse uterus at 18 weeks of age. EGFP immunoreactivity showing embryonic Hh-signal-responsiveness is observed in the uterine vessel (**B**), myometrium (**C**) and endometrium (**D**). (**E**–**G**) Double immunohistostaining of EGFP stained with Histogreen plus endomucin, αSMA and ERα stained with DAB. Double immunohistostaining analysis confirms that EGFP positive cells are localized in the uterine vessel (**E**; red arrows), myometrium (**F**) and endometrium (**G**). (**B**–**D**) Higher magnification of red boxes in (**A**). These results were observed in several individuals (*n* = 3). ue, uterine epithelium; es, endometrial stroma; e.g., endometrial gland; mm, myometrium; uv, uterine vessel. Scale bars indicate 200 μm (**A**) and 50 μm (**B**–**G**).

Next, to follow up the fate of embryonic Hh-responsive cells in the Wolffian region to adulthood, the uterus was examined at 18 weeks after E13.5 TM induction, and various remarkably labeled uterine tissues were detected (Figure 5). In this lineage tracing assay, we used a *Rosa26*-EGFP mouse strain as Cre indicator allele for double immunostaining of EGFP-reporter in addition to tissue specific markers. The majority of EGFP-reporter positive cells were localized mainly on the uterine mesometrial side (Figure 5B,C), and several labeled cells were also present on the antimesometrial side (Figure 5A,D). Additionally, double immunostaining analysis of EGFP with uterine tissue markers revealed EGFP-labeled cells located in several uterine tissues: the endometrium mesenchyme revealed by ERα immunoreactivity (Figure 5G), the myometrial smooth muscle revealed by SMA immunoreactivity (Figure 5F) and vascular vessels revealed by endomucin immunoreactivity (Figure 5E). These results demonstrated the presence of a long-lived progeny from Wolffian Hh-responsive cells, which can contribute to various differentiated uterine tissues in adulthood.

## 3. Discussion

Irreversible genetic marking based on the mouse Cre-loxP recombination system, provides sufficient information for understanding various developmental processes in vivo [17,27,28,29]. With the use of *Gli1*^CreERT2^ mice line (Hh signal-specific Cre-driver) [21], the current study revealed, for the first time, experimental evidence for the formation of the FRT. Namely, Hh-signal-responding progenitors located in the mesenchyme surrounding WD can contribute to distinct uterine components.

**Figure 6:**
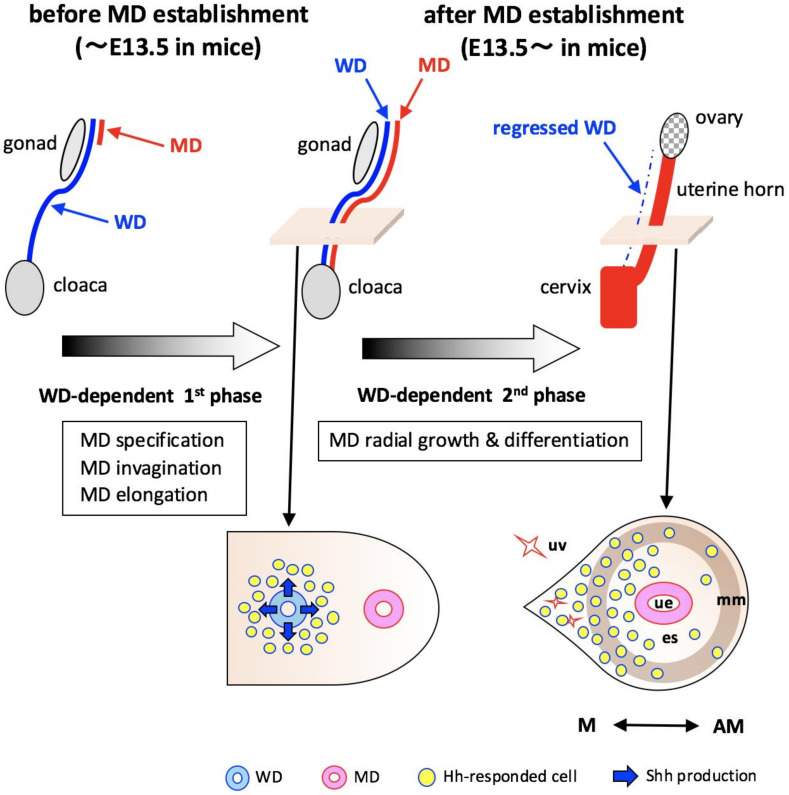
Model showing the developmental mechanism of WD-dependent FRT formation. This schematic model shows that the mouse FRT formation process involves two developmental phases dependent on WDs. The 1st phase (before E13.5 mice): CE cells commit to MD precursors by WD-derived inductive signals. MD precursors then invaginate and elongate caudally within the urogenital ridge towards the cloaca using WDs as “guide wires”. The 2nd phase (after E13.5 mice): after completing MD elongation, radial growth and differentiation of the MD mesenchyme and its surrounding mesenchyme are gradually initiated with the eventual regression of WDs. In this phase, cells within the urogenital ridge respond to the WD-derived Hh signal. These cells showing Hh-signal-responsiveness are localized exclusively around the WDs and continue to exist in the developing uterus after the regression of WDs and directly commit to the differentiation pathways of uterine tissue lineages, smooth muscle, endometrial stroma, and vascular vessel, mainly in their mesometrial domain. WD, Wolffian duct; MD, Mullerian duct; ue, uterine epithelium; es, endometrial stroma; mm, myometrium; uv, uterine vessel; M, mesometrial side; AM, antimesometrial side.

Most of the FRT originates from MDs during embryogenesis [1,2,3]. Recent advances in the research field of FRT development define several cellular and molecular mechanisms for the formation of MDs (Figure 6: WDs-dependent 1st phase). Especially, the discovery of the strong dependency of early MD formation on WDs provides significant insights [6,7,8,9]. The early stage of MD development involves three morphogenetic processes: MD specification, invagination and elongation—processes that are strictly governed by WDs. Collaborative signaling interactions with BMP and FGF on WDs induce MD specification and invagination through the sequential activation of *Pax2* and *Lim1* genes in the coelomic epithelium [6]. During the next phase of MD formation, the elongation process, the direct contact between WDs and MDs is definitely requisite for sustaining caudal migration as “guide wires”, which is supported by the findings that WD-specific gene modulation and cell ablation elicit its degeneration and interruption of MDs elongation [7,8,9].

In this study, we demonstrated that WDs actively participate in the formation of the FRT through Hh signaling, after the establishment of MDs (Figure 6: WDs-dependent 2nd phase). The main morphogenetic event during FRT development after MD elongation, is generally considered tissue interaction between the MD and its surrounding mesenchyme, which is gradually recognizable beginning from mid- or late-embryonic stages [10,11,12]. Here, we found that the major Hh signaling components are expressed mainly in/around WDs at E13.5 mid-stage embryos. In addition, the late-stage *Shh* KO embryos, interestingly, showed a radial growth defect in uterine stroma without any structural destruction of the uterine tube. These results imply that the Hh signal acts in a limited fashion as an WD-derived regulatory factor for the late phase of FRT development.

Moreover, fate mapping with the use of *Gli1*^CreERT2^ mice demonstrated that embryonic urogenital tissues responded to the WD-derived Hh signal and contribute to various uterine tissues. *Shh*, one of the Hh ligands, is expressed only in the WD epithelium within the urogenital ridge in both genders. In female mice, cells showing Hh-signal-responsiveness were localized exclusively around WDs and continued to exist in the developing embryonic uterus after WD regression. After birth, Hh-signal-responsive cells derived from WDs gradually and directly commit to the differentiation pathways of uterine tissue lineages, smooth muscle, endometrial stroma, and vascular vessel, probably through proliferation and migration processes. These findings provide definite evidence that embryonic urogenital tissue under the influence of WD action participate directly in uterine development during embryonic and adult stages. The WD-derived Hh signal might be functionally involved in long-term uterine tissue formation and differentiation. Since *Shh*-null mutation results in embryonic lethality in mice [30], further studies using the WD-specific *Shh* deletion allele [26] are needed to elucidate the functional roles of Hh action in WD-dependent uterine development during postnatal and adult stages.

In conclusion, this is the first study conducted with the use of the *in vivo* cell tracking approach to identify novel lineage contribution to the development of female reproductive organs. The major point in this analysis was that after WD regression, embryonic cells responsive to the WD-derived Hh signal continue to exist in the developing FRT and clearly exhibit lineage contribution to the various tissues of the mouse uterus. Our findings provide new insights into FRT development. Further elucidation by wide-ranging studies on WD-dependent FRT formation would promote full understanding of the regulatory mechanisms underlying fundamental uterine function and/or gynecological diseases of unknown etiology.

## 4. Materials and Methods

### 4.1. Animals

The *Shh* KO and *Gli1*^CreERT2^ mice have been described [21,30]. *Rosa26*-LacZ and *Rosa26*-EGFP (Stock No. 012429) mice were obtained from The Jackson Laboratory [31]. The mice were maintained in a mixed genetic background. All animal experimental procedures and protocols were approved by the Committee on Animal Research at Ehime University (Permit No. 05-KU-34-16, 9 July 2018).

### 4.2. Genetic Lineage Tracing Experiments

Fate mapping analyses focused on Hedgehog-signal-responsive cells were conducted with the use of compound *Gli1*^CreERT2^; *Rosa26*-LacZ or *Gli1*^CreERT2^; *Rosa26*-EGFP mice [20,21,32]. *Gli1*^CreERT2^ male mice were crossed with *Rosa26*-LacZ or *Rosa26*-EGFP homozygous Cre indicator female mice. Stock solutions of Tamoxifen (TM) (Cat. No. T5648, Sigma-Aldrich, St. Louis, MO, USA) were prepared at a concentration of 20 mg/mL in corn oil. TM inducible CreER mediated gene recombination in the embryos (on embryonic day 10.5 or 13.5) was induced by one oral gavage to pregnant mice at 100 mg/kg body weight. To detect Cre-mediated reporter gene expression, mouse tissues were processed for X-gal and immunohistochemical staining with an anti-EGFP antibody. Control experiments were carried out without TM injection (Figure S1).

### 4.3. Histological Analysis

Mouse tissues were dissected and fixed in 4% PFA/PBS for 2 days at 4 °C, dehydrated through ethanol, and embedded in paraffin; 8-μm serial sections were then prepared for histological analysis. Immunohistochemical analyses were carried out by standard procedures [33] using the following antibodies (Ab): anti-EGFP (1:1000, Cat. No. ab183734, Abcam, Cambridge, UK), anti-endomucin (1:500, Cat. No. sc-53941, Santa Cruz, Dallas, TX, USA), anti-SMA (1:10,000, Cat. No. M0851, Dako, Carpinteria, CA, USA), anti-ERα (1:20,000, Cat. No. 1115-1, Epitomics, Burlingame, CA, USA), anti-E-cadherin (1:500, Cat. No. 3195, Cell Signaling Technology, Danvers, MA, USA), and anti-CD31 (1:100, Cat. no. ab28364, Abcam, Cambridge, UK). HRP-conjugated anti-rabbit IgG (Cat. No. K4003, Dako, Carpinteria, CA, USA) or anti-rat IgG (Cat. No. MP-7404, Vector Laboratories, Burlingame, CA, USA) was used for the detection of primary antibodies. Signal detection was carried out with 3,3′-diaminobenzidine (DAB, Cat. No. K3468, Dako, Carpinteria, CA, USA) and 3,3′-diaminobenzidine tetrahydrochloride dihydrate (HistoGreen, Cat. No. E109, Eurobio-Abcys, Courtaboeuf, France). Section in situ hybridization for gene expression was analyzed, and the antisense riboprobes were prepared for Shh, Ptch1, Gli1, Gli2, and Gli3, as described [20,34,35]. Sections were hybridized with digoxigenin (DIG)-labeled antisense riboprobes and immune-detected with alkaline phosphatase conjugated anti-DIG antibody (Cat. No. 11093274910, Roche, Basel, Switzerland). Signals were detected with NBT/BCIP substrate (Cat. No. 11383213001/11383221001, Roche, Basel, Switzerland).

### 4.4. X-Gal Staining

LacZ reporter gene expression was detected as previously described [20,33]. Dissected tissues were fixed in 0.8% paraformaldehyde (PFA) and 0.02% glutaraldehyde in PBS for 2 days at 4 °C and washed five times in PBS before X-gal staining by standard procedures. X-gal stained tissues were rinsed in 70–100% ethanol, embedded in paraffin wax, sectioned at 6 μm, counterstained with eosin, and immunohistochemically stained.

## Supplementary Materials

Supplementary materials can be found at https://www.mdpi.com/1422-0067/22/3/1211/s1.
