# Supplementary material for: New Insights into Development of Female Reproductive Tract—Hedgehog-Signal Response in Wolffian Tissues Directly Contributes to Uterus Development
Source: Int J Mol Sci. 2021 Jan 26;22(3):1211. doi: 10.3390/ijms22031211 (PMC7865753; doi:10.3390/ijms22031211)
Supplement: Supplementary file 1 [file ijms-22-01211-s001.pdf]

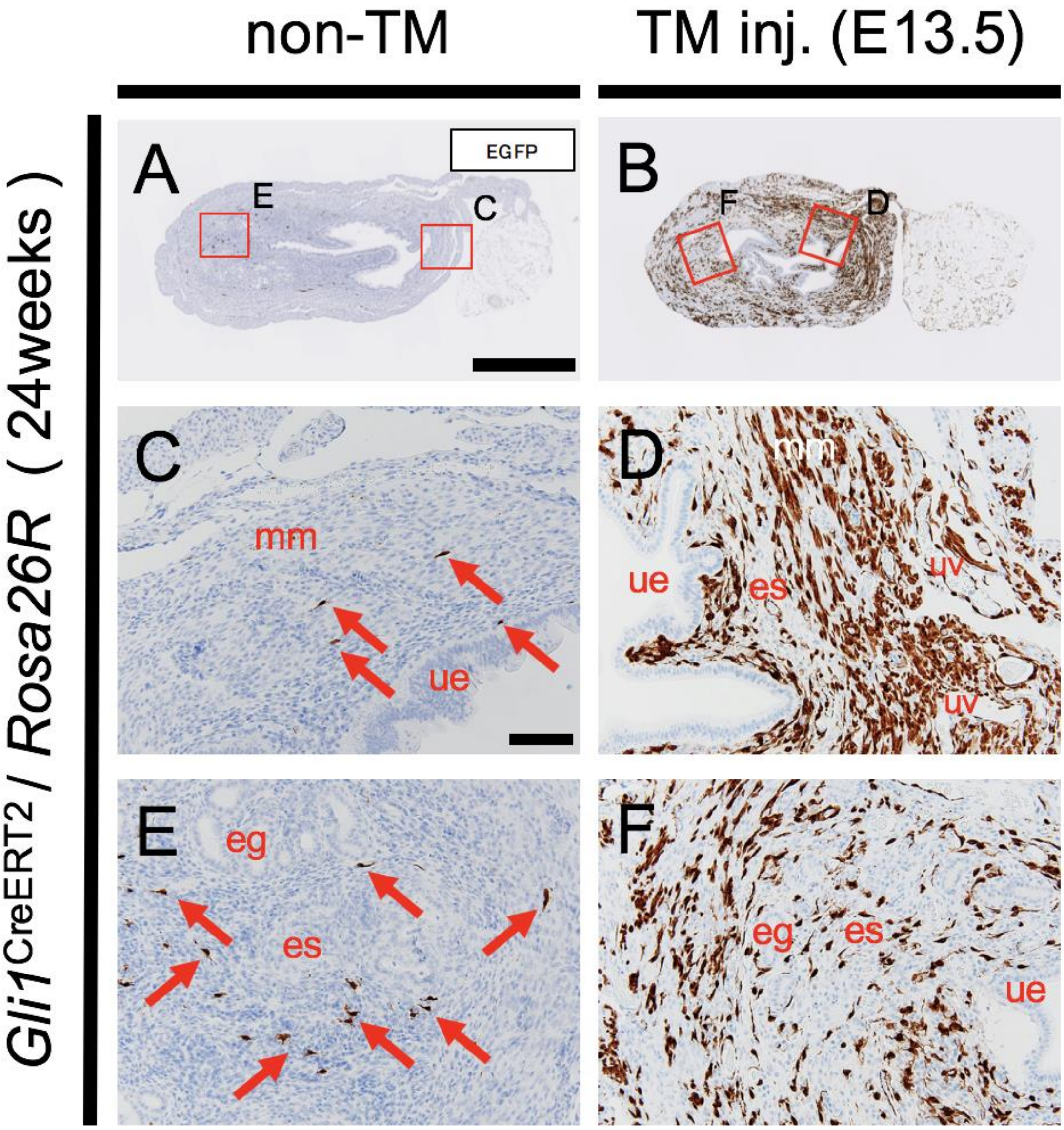

Figure S1. Inducible CreER activity under the control of *Gli1*^{CreERT2} mouse line allows the manipulation of gene activity during uterine formation. (A-F) Immunohistostaining with anti-EGFP Ab of the mouse uterus at 24 weeks of age. (A, C, E) Without tamoxifen (TM) injection, very low EGFP immunoreactivity is observed in uterine tissues, indicating the leaky CreER activity of *Gli1*^{CreERT2} mouse line (Red arrows in C and E). In contrast, E13.5 TM administration led to a dramatic increase in the number of EGFP-labeled embryonic Hh-responsive cells in various uterine tissues, indicating efficient activation of CreER by TM. (B, D, F). Leaky *Gli1*^{CreERT2} activity without TM injection (A, C, E) do not significantly influence the interpretation of the results in our fate mapping analysis. (C-F) Higher magnification of red boxes in (A) and (B). ue, uterine epithelium; es, endometrial stroma; eg, endometrial gland; mm, myometrium; uv, uterine vessel. Scale bars indicate 1 mm (A, B) and 100 m (C-F).

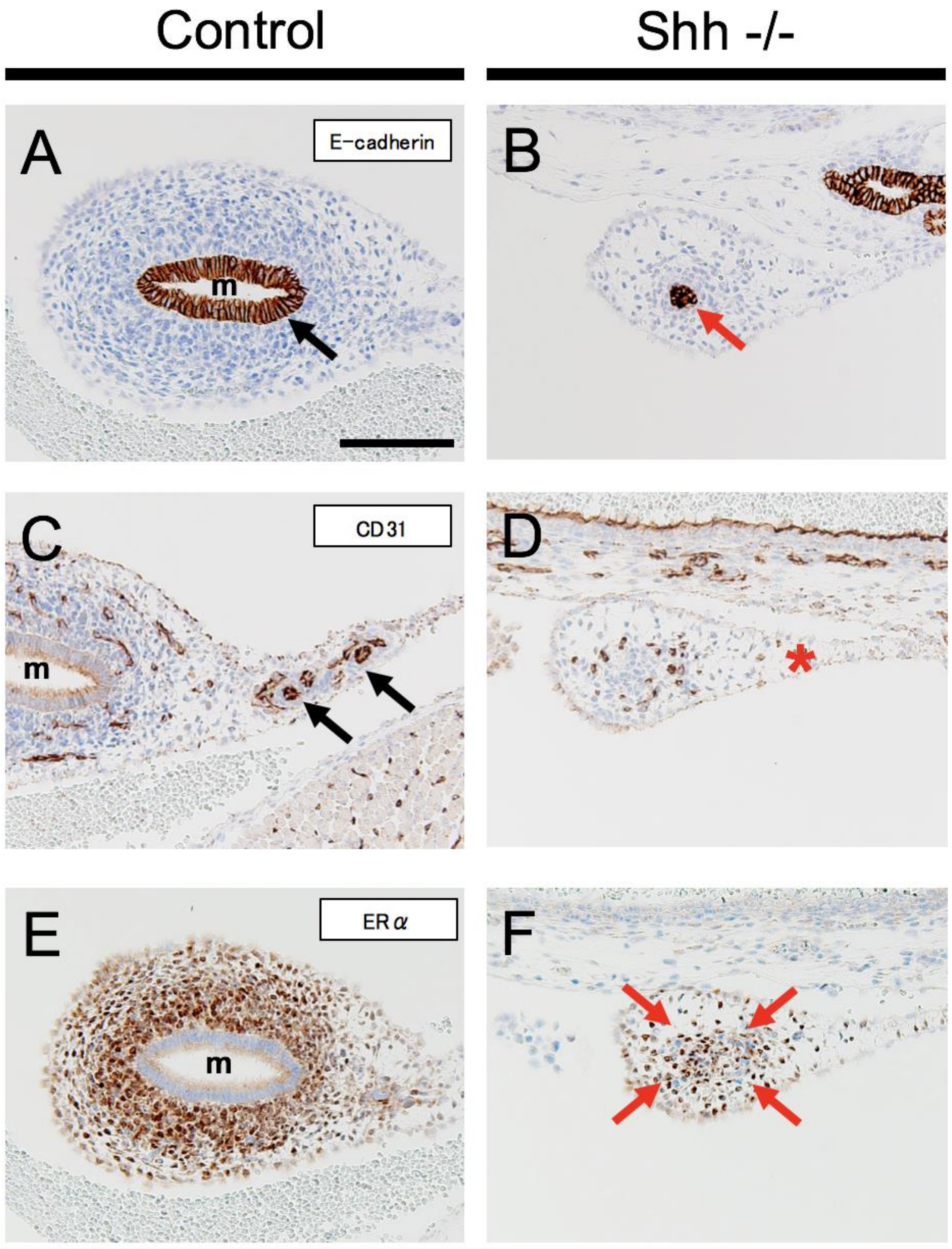

Figure S2. Histological molecular characterization of *Shh* mutant female mice uteri. (A-F) Sections immunostained with anti-E-cadherin Ab, anti-CD31 Ab and anti- ER Ab of the mice uterus at newborn stage. (A, B) A marked E-cadherin (epithelial cells marker) protein expression is observed in both control and *Shh* mutant mice. The mutant mullerian duct (Red arrows in B) was barely recognizable as tubular structure compared with that in control mice (Black arrows in A). (C, D) CD31 (endothelial marker) protein expression in control and *Shh* mutant mice uterus. Mutants indicate the lack of CD31 positive uterine artery (shown by asterisk in D) compared with that in

control mice (Black arrows in C). (E, F) Compared with control mice uterus, a significant decrease of ER (uterine stromal cell marker) protein expression is observed in mutants. Mutants showed a prominent reduction of ER immunoreactivity in the mesenchymal mass surrounding the mullerian duct (Red arrows in F). m, mullerian duct. Scale bars indicate 100 m (A-F).
